# The Different Functional Distribution of “Not Effector” T Cells (Treg/Tnull) in Colorectal Cancer

**DOI:** 10.3389/fimmu.2017.01900

**Published:** 2017-12-22

**Authors:** Elena Niccolai, Federica Ricci, Edda Russo, Giulia Nannini, Giacomo Emmi, Antonio Taddei, Maria Novella Ringressi, Filippo Melli, Manouela Miloeva, Fabio Cianchi, Paolo Bechi, Domenico Prisco, Amedeo Amedei

**Affiliations:** ^1^Department of Surgery and Translational Medicine, University of Florence, Florence, Italy; ^2^Department of Biomedical, Experimental and Clinical Sciences “Mario Serio”, University of Florence, Florence, Italy; ^3^Department of Clinical and Experimental Medicine, University of Florence, Florence, Italy

**Keywords:** colorectal cancer, tumor-infiltrating lymphocytes, regulatory T cells, not effector T cells, T helper, tumor microenvironment, antitumor immune responses

## Abstract

Colorectal cancer (CRC) is the third most common cancer worldwide, ranking as high as the second leading cause of cancer-related deaths in industrialized countries. Consistent with immunosurveillance theory, the immune system is crucial to protect the host from developing tumors, and the major players in tumoral immunity are effector T cells. Anyway, cancer cells develop strategies of immunoevasion influencing the cancer-specific lymphocyte priming, activation, and effector function. Therefore, the T cell subsets that mature during the stages of tumor growth, differently contribute to disease progression and/or regression. In our study, we analyzed the intra-tumoral and peripheral T cell subsets’ distribution in 30 patients with CRC, in order to clarify their functional role toward cancer. We found that percentage of infiltrating effector T cells decreased in cancer tissue than in healthy mucosa and that the tumor microenvironment negatively influences the cytolytic activity of T lymphocytes reactive to cancer cells. Moreover, we found that the tumor tissue was infiltrated by a large amount of “not effector” T (neT) cells with a regulatory or an anergic profile, which are unable to kill cancer cells, may be contributing to the CRC promotion. The presence of neT cells was investigated also in the peripheral blood of CRC patients, demonstrating that the peripheral T regulatory cells can inhibit the proliferation of effector T cells, confirming their immunosuppressive properties. Finally, monitoring the changes in circulating neT cells’ frequencies after the tumor removal, we confirmed the role of cancer in the modulation of immune system, in particular, in supporting a Tregs-mediated immunosuppression.

## Introduction

Colorectal cancer (CRC) is the third most common cancer worldwide, ranking as high as the second leading cause of cancer-related deaths in industrialized countries (1). Colorectal carcinogenesis represents a heterogeneous process with a differing set of somatic molecular alterations, influenced by diet, environmental/microbial exposures, and host immunity. CRC usually begins as benign polyps, sometimes referred as pre-cancerous, i.e., adenomatous polyps and evolves through the stepwise accumulation of genetic/epigenetic alterations; so, the genotypic variations have a high impact, but in addition to some host factors, such as the cells of the immune response.

Consistent with immunosurveillance theory, the immune system is important to protect the host from developing tumors (2), but that the cancer cells can develop immunoevasion strategies (3) and also, the immune cells can act, supporting the tumor progression (4). The major players of adaptive immunity are effector T cells, but the tumoral immunoevasion mechanisms appear to influence diverse steps in cancer-specific lymphocyte priming, activation, and effector function (5). Therefore, the T cell subsets that develop during the stages of cancer growth, differently contribute to disease progression and/or regression. In particular, CD45RO^+^ memory T lymphocytes, cytotoxic CD8^+^ T cells (CTLs) and interferon (IFN)-γ-producing T helper1 cells (Th1) have been found to be associated with prolonged survival in CRC, irrespective of tumor stage (6–8), while the role of Th2 cells in colon cancer is mainly harmful as IL-4 directly and indirectly favors tumor growth (9–11). CD4^+^ T cells are now known to differentiate into additional effector T-cell subsets (i.e., Th17, Th9, follicular helper T, Th22) with contrasting and unclear roles in the development of CRC (12–14) as well as immunosuppressive cells, like exhausted, anergic/tolerant, and subsets of regulatory T cells (Tregs) that can favor cancer progression (15–18). In fact, the CD4^+^ CD25^+^ Foxp3^+^ Tregs subpopulation may exert a critical role in cancer promotion (19, 20).

Regulatory T cells are essential in the maintenance of immune homeostasis as illustrated by spontaneous autoimmune disease development when Tregs are rendered deficient (21, 22). Conversely, it is now well verified that a large number of Tregs infiltrate the tumor tissues of different cancers (breast, lung, pancreas, and ovary) (23–26) and their abundant presence is often associated with poor clinical prognosis and negatively correlated with patients’ survival (27). Notably, decreased ratios of tumor-infiltrating CD8^+^ T cells to FOXP3^+^ Tregs were shown to correlate with poor prognosis, especially in patients with breast (28), gastric (18), and ovarian cancer (29). By contrast, reports show that a high intra-tumoral frequency of Tregs is correlated with a good prognosis, in patients with Hodgkin lymphoma or CRC (10, 19, 30–32). These conflicting results may arise from an improper interpretation of the heterogeneous FOXP3^+^ cells (i.e., “effector” Tregs and non-Tregs) (33) as a single population of Tregs.

Thus, it is critically important to assess the functional properties of Tregs in the tumor tissues in order to evaluate their contribution to anticancer immune response.

In this study, we have characterized the tumor-infiltrating lymphocytes’ subsets in CRC patients to clarify their protective or favoring role toward cancer. The presence of anti-tumoral T cells or not effector T (neT) cells was investigated also in the peripheral blood of the same CRC patients, in order to evaluate if the peripheral T cell distribution mirror the intra-tumoral immune response. Finally, we dynamically investigated the changes in circulating Tregs’ frequencies after the tumor removal to assess the impact of cancer cells in the induction of Tregs.

## Materials and Methods

### Patients

Thirty patients with CRC (18 males and 12 females, mean age 67.3 years) and 30 healthy controls were enrolled after obtaining informed consent and approval of the local ethical committee (*Comitato Etico Area Vasta Centro*). Specifically, CRC patients’ characteristics are summarized in Table 1. Cancer samples were classified as colorectal adenocarcinoma according to the TNM classification of colorectal tumors. All patients underwent surgical resection of the primary lesion but did not receive chemotherapy; patients with evidence of serious illness, immunodeficit, or autoimmune or infectious diseases were excluded. As healthy controls, we enrolled asymptomatic patients, matched with respect to age and sex.

**Table 1:** Clinical characteristics of colorectal cancer (CRC) patients: T cell clones (Tcc) generated and pre/postoperative values of circulating CD4^+^ regulatory T cells (Tregs).

Code	CEA (mean ± SD)	TNM	Tcc obtained	Number of intra-tumoral Tregs	Preoperative values of Tregs	Postoperative values of Tregs
Crc 01	4.98 ± 1.48	pT3 NO Mx	21	1	2.4	1.9
Crc 02	3.69 ± 1.82	pT3 NO Mx	11	6	6.3	4.6
Crc 03	3.65 ± 2.12	pT3 N2a Mx	17	4	4.2	3.4
Crc 04	3.50 ± 1.65	pT3 NO Mx	15	6	5.4	4.2
Crc 05	4.28 ± 1.89	pT3 N1B Mx	20	4	4.5	3.6
Crc 06	4.30 ± 2.74	pT2 NO Mx	10	1	2.6	2.1
Crc 07	3.90 ± 1.65	pT3 N2a Mx	21	2	3.4	2.9
Crc 08	2.50 ± 1.43	pT2 NO Mx	17	3	3.5	1.9
Crc 09	4.37 ± 2.35	pT3 N2a Mx	15	2	2.9	2.2
Crc 10	4.25 ± 1.89	pT2 NO Mx	12	3	3.9	3.2
Crc 11	4.50 ± 2.38	pT3 N2a Mx	32	3	3.7	3.1
Crc 12	3.50 ± 2.62	pT2 NO Mx	24	2	3.8	2.8
Crc 13	4.36 ± 1.72	pT3 N2a Mx	27	6	4.5	3.5
Crc 14	4.20 ± 1.69	pT2 NO Mx	35	0	2.3	1.9
Crc 15	3.90 ± 2.15	pT3 NO Mx	30	1	3.4	2.5
Crc16	2.50 ± 1.62	pT3 NO Mx	38	1	3.6	2.8
Crc 17	5.09 ± 2.33	pT2 NO Mx	41	1	2.7	1.5
Crc 18	4.90 ± 2.63	pT2 NO Mx	27	1	3.4	1.9
Crc 19	4.90 ± 1.56	pT3 N2a Mx	25	2	3.5	2.8
Crc 20	3.50 ± 1.68	pT3 N2a Mx	36	1	2.8	2.1
Crc 21	4.95 ± 2.12	pT3 NO Mx	31	4	4.3	3.5
Crc 22	3.93 ± 2.63	pT3 NO Mx	26	1	2.9	2.2
Crc 23	4.20 ± 1.84	pT3 N2a Mx	28	2	3.7	2.9
Crc 24	3.80 ± 2.32	pT3 N2a Mx	32	1	2.4	1.5
Crc 25	2.66 ± 2.72	pT2 NO Mx	30	5	4.1	3.6
Crc 26	4.58 ± 1.98	pT2 NO Mx	30	3	3.8	3.1
Crc 27	4.90 ± 1.85	pT3 N2a Mx	26	1	3.2	2.1
Crc 28	4.62 ± 1.98	pT3 N2a Mx	37	0	2.8	1.8
Crc 29	4.36 ± 2.62	pT3 N2a Mx	25	2	3.6	3.1
Crc 30	2.50 ± 1.62	pT3 NO Mx	42	1	3.7	2.9
All CRC patients	4.04 ± 2.36	Colorectal adenocarcinoma	781	70	3.58 ± 0.88	2.72 ± 0,79

*CEA, antigencarcinoembrionic*.

### Generation of T Cell Clones (Tcc) from TILs of the Neoplastic Colonic Tissue

Surgical specimens of CRC tissue were dissociated in order to isolate tumor-infiltrating lymphocytes (TILs). In particular, tissue pieces from each patient were obtained from three different sites, namely: central tumor (CT), marginal tumor (MT), and surrounding healthy mucosa (HM). First, tissue pieces were dissociated with the Tumor Dissociation Kit, human (Miltenyi Biotech, UK) in combination with the gentleMACS™ Octo Dissociator (Miltenyi Biotech, Germany), to obtain a gentle and rapid generation of single-cell suspensions. Then, TILs were magnetically isolated with anti-human CD3 microbeads (Miltenyi Biotech, UK) and cloned under limiting dilution, as described previously (34). Briefly, ingle T-cell blasts were seeded in microwells (0.3 cells/well) in RPMI 1640 culture medium (SERO-Med GmbH, Wien) supplemented with 10% FCS HyClone (Gibco Laboratories, Grand Island, NY), in the presence of 2 × 10^5^ irradiated (9,000 rad) peripheral blood mononuclear cells (PBMC), phytohemagglutinin (0.5% vol/vol), and recombinant human interleukin-2 (IL-2) (50 U/ml) (PeproTech, USA). At weekly intervals, 2 × 10^5^ irradiated PBMC and IL-2 were added to each micro-culture to maintain the expansion of growing clones. The Tcc were evaluated for their surface markers and functional properties: in detail cytokine profile and cell cytotoxicity.

### Phenotypic and Functional Characterization of Isolated Tcc

We analyzed the Tcc’ surface markers (CD3, CD4, CD8, CD25, CCR7, CD45RA, CD45 RO, and CD28) expression by flow cytometry using a BD FACScan cytofluorimeter as previously described (34). A total of 10^4^ events for each sample was acquired. To assess the cytokines’ production Tcc were resuspended at a concentration of 10^6^ cells/ml medium and cultured for 36 h in the presence of PMA (10 ng/ml) plus ionomycin (200 ng/ml). Cell-free supernatants were collected and assayed in duplicate for IFN-γ, IL-4, and IL-17, content by commercial ELISA kits (Bio-Source International, Camarillo). The supernatants presenting cytokine levels that were 5 SD above the mean levels in control supernatants derived from irradiated antigen-presenting cells alone were regarded as positive. Based on the cytokine profile and the CD4/CD8 expression, we divided the Tcc into the following groups: Th1/Tc1 (only IFN-γ), Th2/Tc2 (only IL-4), Th0/Tc0 (IL-4 + IFN-γ), and Th17/Tc17 (IL-17). Tcc unable to produce IFN-γ, IL-4, and IL-17 were analyzed by flow cytometry for FoxP3, TGF-beta, and IL-10 markers’ expression. Fluorochrome-conjugated anti-human (IL-10, TGF-β, and FoxP3) mAbs were purchased from eBioscience (San Diego, CA, USA). For intracellular analysis, the blasts were stimulated using the Leukocyte Activation Cocktail, with BD GolgiPlug™ (BD Pharmingen), and stained with anti-IL-10, anti-TGF-β, and anti-FoxP3 mAbs. For the FoxP3 detection, Tcc were fixed and permeabilized using the BD Pharmingen Human FoxP3 Buffer Set (BD Biosciences). Tcc that were negative for FoxP3, IL-10, and TGF-β were defined as Tnull, and Tcc that were positive for FoxP3, IL-10, and TGF-β were defined as Tregs.

### Flow Cytometric Detection of Granzyme A

Granzyme A expression by Tcc was assessed according to the manufacturer’s instructions (BD Biosciences, NJ, USA). The different subpopulations of Tcc were tested for their ability to kill P815 target cells upon anti-CD3 mAb activation (5 µg/ml) by evaluating the percentage of AnnexinV-binding cells by flow cytometry and using the FACSDiva software.

### Analysis of Peripheral and Intra-Tumoral T Cell Subsets in CRC Patients

For CRC patients, heparinized venous blood samples were collected on the day of the surgery (*T* = 0) and 2 weeks after (*T* = 14) the removal and PBMCs were isolated by density gradient centrifugation. By flow cytometry, TILs isolated from dissociated tumor tissue and PBMC samples were characterized for the expression of CD4, CD8, CD25, CD127, IFN-γ, IL-4, IL-17, and FoxP3 using intracellular cytokine staining. Briefly, fresh PBMCs were cultured in RPMI 1640 culture medium (SERO-Med GmbH, Wien) supplemented with 10% FCS HyClone (Gibco Laboratories, Grand Island, NY, USA) and stimulated for 5 h using the Leukocyte Activation Cocktail, with BD GolgiPlug™ (BD Pharmingen). Cells were first stained for the surface antigens, then fixed with 4% (w/v) paraformaldehyde and permeabilized with 0.5% saponin, followed by intracellular staining with anti-IL-4, anti-IL-17, and anti-IFN-γ mAbs (BD Biosciences). For the detection of peripheral Tregs, PBMC were fixed and permeabilized using the BD Pharmingen Human FoxP3 Buffer Set (BD Biosciences). A minimum of 10,000 events was acquired for each test and data were analyzed using DIVA software. Based on the cytokine profile and the extracellular markers expression, we identified the following T cell subsets: Th1/Tc1 (only IFN-γ), Th2/Tc2 (only IL-4), Th0/Tc0 (IL-4 + IFN-γ), Th17/Tc17 (IL-17), and Treg/Tcreg (CD25^+^, CD127^−^, FoxP3^+^) (35). We defined as Tnull/Tcnull the percentage of CD4^+^/CD8^+^ T cell that did not produce any of the tested cytokine. PBMCs were collected also from the healthy controls, in order to compare the Tregs and Tnull distributions with those of CRC patients.

### Purification and Functional Evaluation of Blood Tregs

To assess the suppressive properties of Tregs isolated from the peripheral blood of CRC patients, we tested their ability to inhibit the proliferation of T effector cells (Teff), as previously described (36). Briefly, peripheral Tregs were magnetically separated from CRC patients’ PBMC, using the CD4^+^CD25^+^CD127^dim/−^ Regulatory T Cell Isolation Kit (Miltenyi Biotec, Germany): cells eluted from the column constituted Tregs, whereas T cells not retained by the magnetic field were accounted as Teff. Tregs and Teff were cultured alone or together at a 1:1 ratio in complete medium, with or without polyclonal stimulation [1 µg/ml, low-endotoxin anti-CD3 and anti-CD28 monoclonal antibodies (BD Biosciences)]. The stimulation was carried out immediately after BD Horizon Violet Proliferation Dye 450 (VPD450, BD Biosciences) incorporation. The proliferation of Teff, alone or in the presence of Tregs, was assessed after 5-day incubation by flow cytometry.

### Statistical Analysis

The analysis was performed using SPSS statistical software (version 24) and the data were expressed as the mean ± SD for continuous variables. Comparisons between the two groups were assessed using the Student’s *t*-test. Correlations between the parameters were assessed using Pearson correlation analysis. One-way ANOVA test was performed to compare three or more groups with Turkey’s multiple comparison test. SPSS was used for all the statistical analyses. A multiple regression analysis was performed to correlate Tregs percentage values and clinical parameters. *p* Values of less than 0.05 were considered significant.

## Results

### Characterization of CRC Tumor-Infiltrating Lymphocytes (TILs)

**Figure 1:**
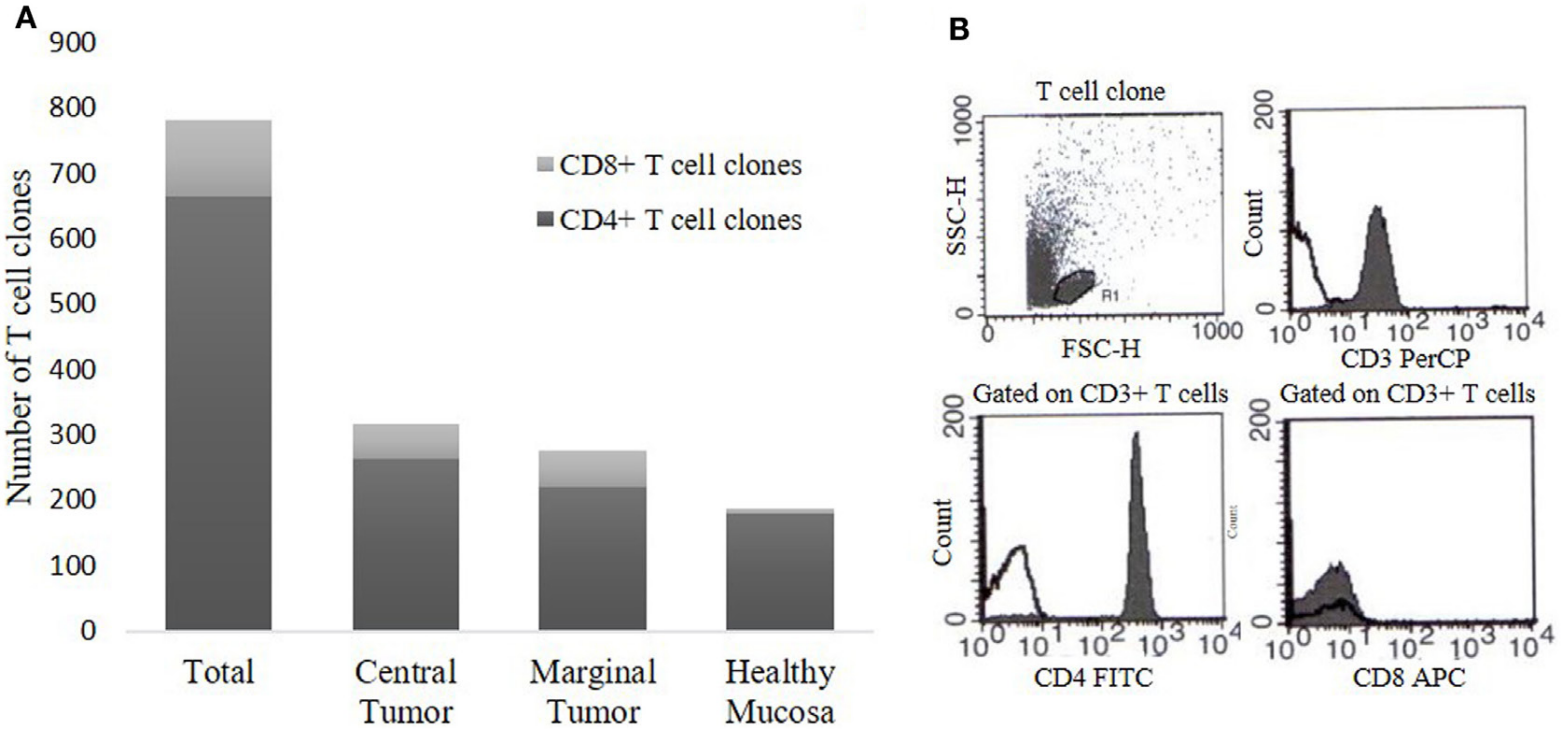
T cell clones (Tcc) obtained from colorectal cancer (CRC) patients. **(A)** The histogram represents the number of CD4^+^ and CD8^+^ Tcc isolated from the three different sites, namely central tumor, marginal tumor, and surrounding healthy mucosa. **(B)** A representative flow cytometry analysis of a CD4^+^ Tcc: anti-CD3, anti-CD4, anti-CD8 mAb (filled curve), or isotype control (empty curve).

To evaluate the intra-tumoral immune response in CRC patients, we expanded and cloned *in vivo*-activated TILs, isolated from three different sites (CT, MT, and HM). We obtained Tcc from each patient (Table 1) for a total number of 781 Tcc: taking into account all patients, the number of Tcc obtained (529) from the tumor tissue (CT + MT) was higher than those obtained from HM (252). In detail, we have isolated 315 Tcc from the tissue of CT and 214 from the MT. Eighty-five percent (664/781) of isolated Tcc were positive for CD4 with a similar distribution on the evaluated cancer tissues: 264, 220, and 180 from CT, MT, and HM, respectively. Noteworthy, of the obtained 117 CD8^+^ Tcc, only seven (7/117, 6%) were isolated from the HM, the majority (94%) was isolated from the tumor tissue (54 from CT and 56 from MT) (Figure 1).

**Figure 2:**
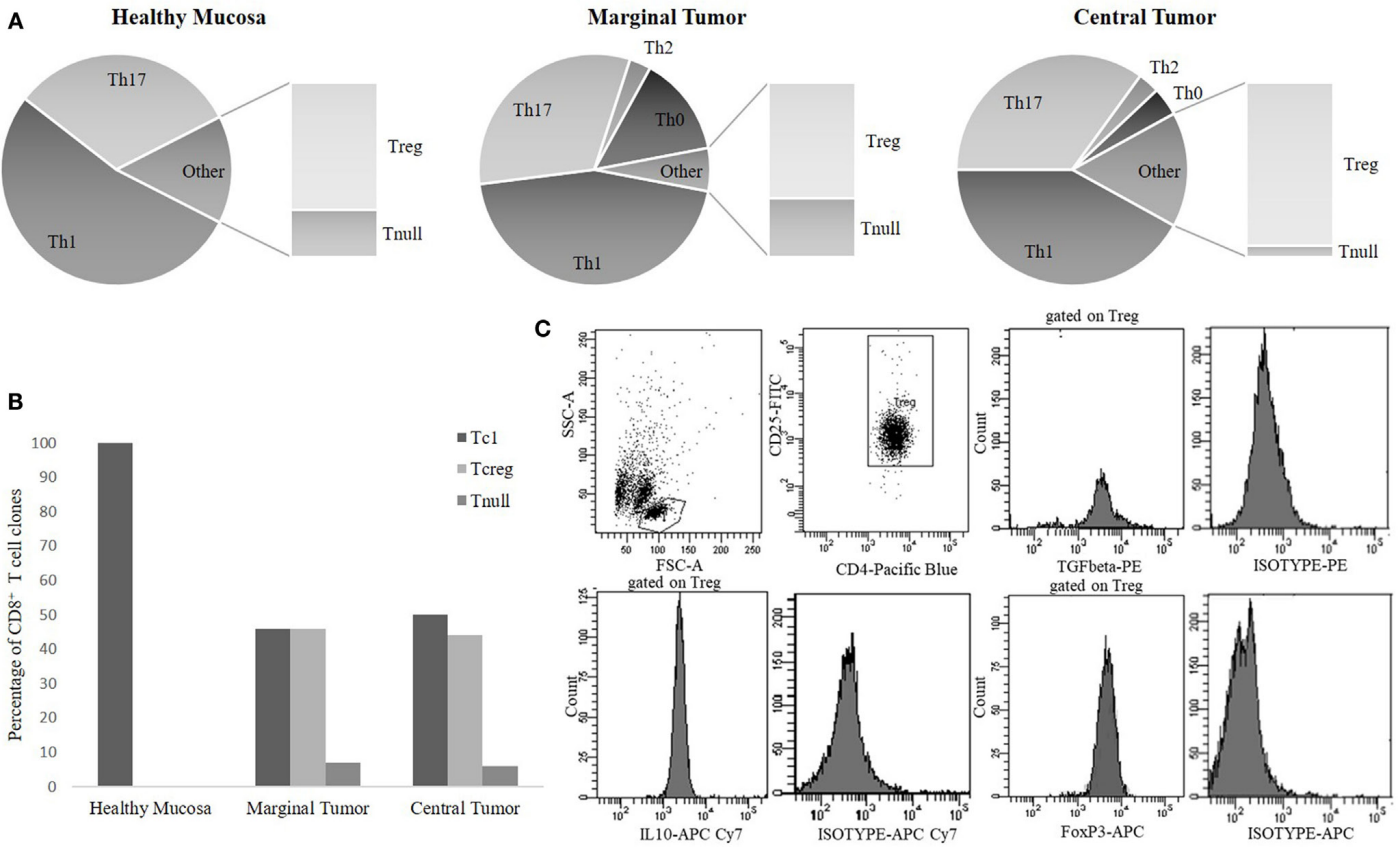
The cytokine phenotype distribution of CD4^+^ T-cell clones **(A)** and CD8^+^ T-cell clones **(B)**. The percentages of CD4^+^ [T helper1 cells (Th1), Th17, Th0, Treg, Tnull] and of CD8^+^ (Tc1, Tcreg, Tnull) T cell clones (Tcc) subpopulations were calculated comparing the number of Th/Tc types with the total number of CD4/CD8 clones obtained from the same tumor tissue sites [healthy mucosa (HM), marginal tumor (MT), and central tumor (CT)]. **(C)** A representative flow cytometry analysis of a CD4^+^ Treg clone: anti-CD4, anti-CD25, anti-FoxP3, anti-TGFβ, anti-IL-10 mAb, and isotype controls.

Based on the cytokine profile and the CD4/CD8 expression, most of the CD4^+^Tcc (306/664, 46%) was Th1 with a similar percentage distribution into the different tissue sites: 96/180 (53%) in HM, 99/220 (45%) in the MT, and 111/264 (42%) in the CT (Figure 2A). A considerable fraction (219/664, 33%) of the CD4^+^ Tcc was able to produce IL-17, in detail, 57 out of 180 Tcc (32%) isolated from HM, 70 of 220 (32%) from MT, and 92 out of 264 (35%) obtained from the CT (Figure 2A). It is remarkable that 62% (136/219) of Th17 clones produced both IL-17 and IFN-γ (data not shown). 13 of the remaining CD4^+^ Tcc produced IL-4 alone: 6 from the MT (3%) and 7 from the CT (3%). Forty CD4^+^ Tcc showed a Th0 profile: 30 from the MT (14%) and 10 from the CT (4%) (Figure 2A). Of note, the Tcc with Th0 or Th2 profile have only been isolated from cancer tissue (marginal + CT). Finally, 86 CD4^+^ Tcc (13%) was unable to produce any of the tested cytokines [not effector T cell clones (neT Tcc)].

Regarding the cytokine profile of the CD8^+^ population, we found that the majority of these Tcc have been isolated from the cancer tissue (110) and that 53 showed a Tc1 profile (producing IFN-γ), in detail 26/56 (46%) from MT and 27/54 (50%) from CT. Finally, all the seven CD8^+^ Tcc isolated from HM secreted only IFN-γ, showing a Tc1 profile (Figure 2B). The 49% of CD8^+^ Tcc did not produce the tested cytokines (neT Tcc).

**Figure 3:**
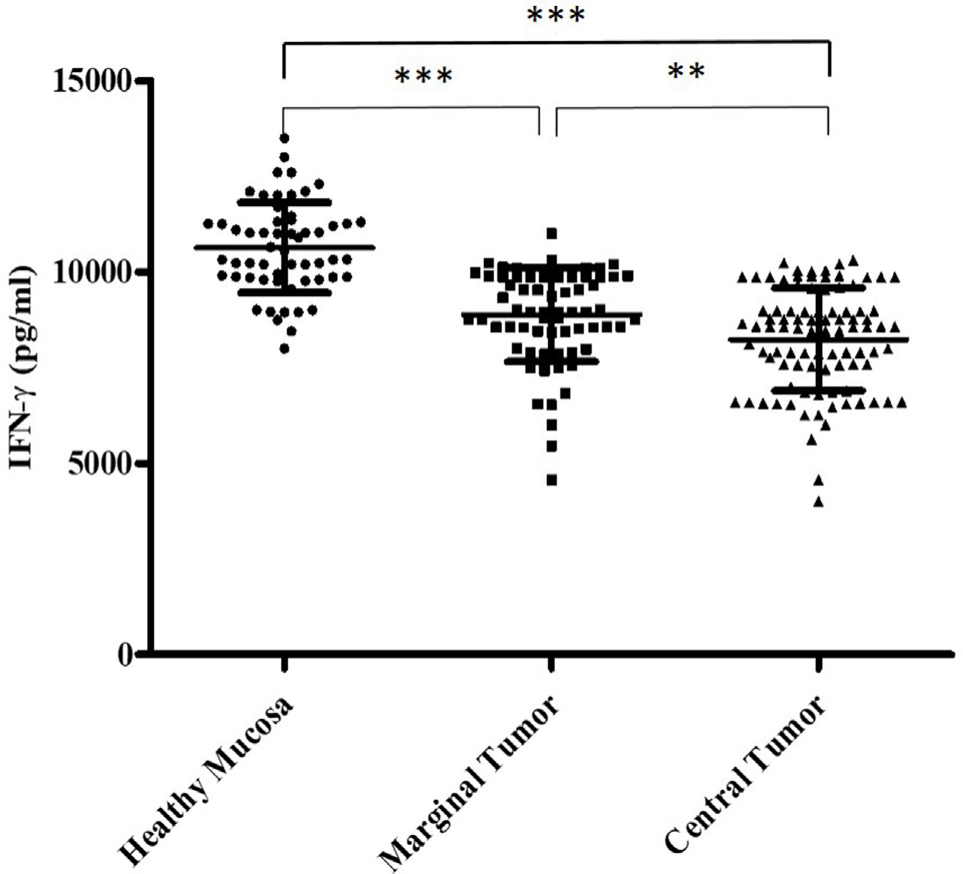
The different interferon (IFN)-γ amount (pg/ml) secreted by the T cell clones with T helper1 cells (Th1) profile, isolated from the tumor tissue. We measured the interferon (IFN)-γ production in culture supernatants by specific test ELISA. Th1 cell clones isolated from healthy mucosa significantly produced higher IFN-γ amount compared to that produced by Th1 cell clones generated from both marginal and central tumor. The error bars represent the SD. Statistical analyses were calculated using one-way ANOVA and Turkey’s *post hoc*. The asterisks (*) represent *p*-values, *p < 0.05, **p < 0.01, ***p < 0.001.

In addition, we have evaluated the IFN-γ amount secreted by the Tcc with Th1 profile isolated from the three different tumor sites. We learnt that the Th1 isolated from HM significantly (*p* < 0.0001) produced higher IFN-γ amount compared to that produced by Th1 Tcc generated from both marginal and CT (Figure 3).

### Intra-Tumoral Tregs in CRC Patients

Evaluating the T cell cytokine profile, we found that a high number (86/664) of CD4^+^ (13%) and 57 of 117 CD8^+^ (49%) T cells was unable to produce any of the tested cytokines, namely neT Tcc. To clarify the nature of these Tcc we subsequently analyzed FoxP3 expression, along with TGF-β and IL-10 production.

Seventy of the 86 CD4^+^ (81%) neT Tcc isolated were FoxP3^+^ and produced IL-10 alone or in combination with TGF-β, showing a definite Tregs profile (Figure 2C), while we defined the remaining 16 (19%) of them as Tnull, because were FoxP3^−^ and unable to secrete both IL-10 or TGF-β. In detail, we have isolated 20 Tregs from HM and 50 from the tumor site (40 CT and 10 MT). About the Tnull population, we have obtained 7 Tcc from the HM and 5 and 4 Tcc from marginal and CT, respectively. Therefore, we have isolated Tnull and Tregs at both the sites. The Tregs’ percentage is higher in the CT (15%) than in HM (11%), and the percentage of Tnull is slightly higher in HM (4%) than in CT (2%), and MT (2%) (Figure 2A).

Regarding the 57 CD8^+^ neT Tcc, the majority (50, 88%) showed a Tcreg profile (FoxP3^+^ and IL-10^+^) and only 7 (12%) were FoxP3^−^ and unable to secrete IL-10, namely Tcnull. In detail, of the 54 CD8^+^ Tcc isolated from the CT 24 (44%) showed a regulatory profile while three (6%) were Tcnull. Similarly, from the MT, we have obtained 26 Tcreg (46%) and four Tcnull (7%) (Figure 2B).

**Figure 4:**
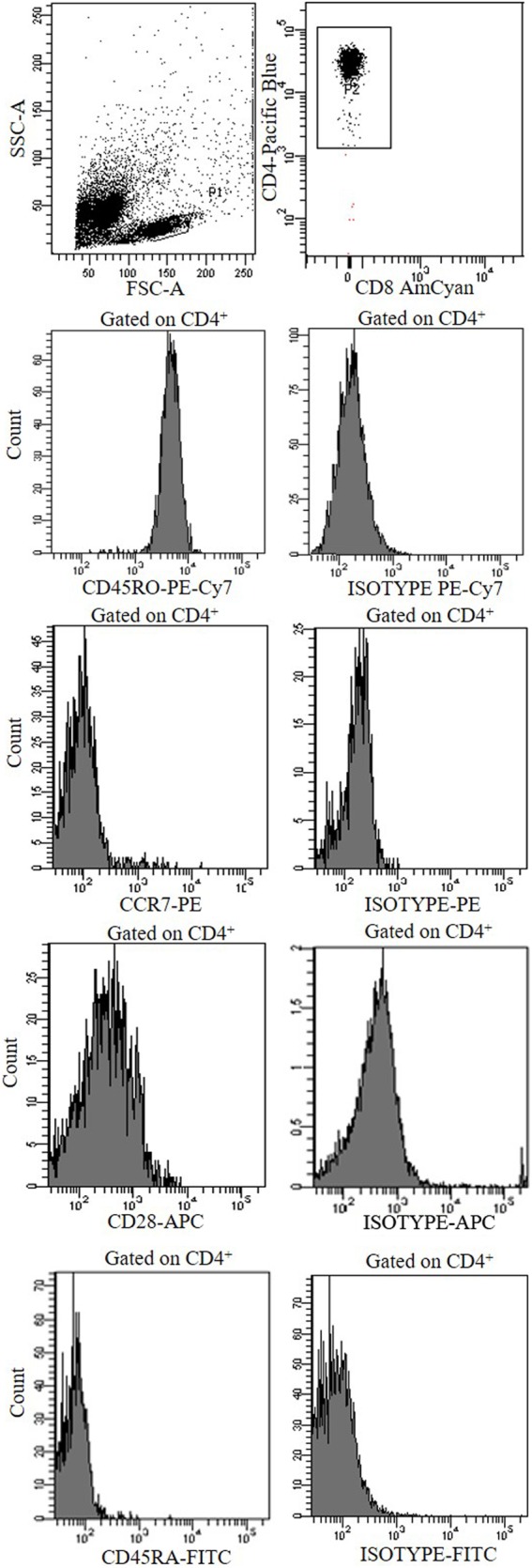
A representative cytofluorimetric analysis (CCR7, CCD28, CD45RO, CD45RA, and isotype controls) of a CD4^+^ Tnull clone obtained from the central tumor of a colorectal cancer patients.

To rule out the possibility that Tnull/Tcnull clones were naive T cells, we have analyzed the expression of CCR7, CD45RA, CD45 RO, and CD28 confirming their memory phenotype (Figure 4).

### Functional Profile of Intra-CRC Tcc

**Figure 5:**
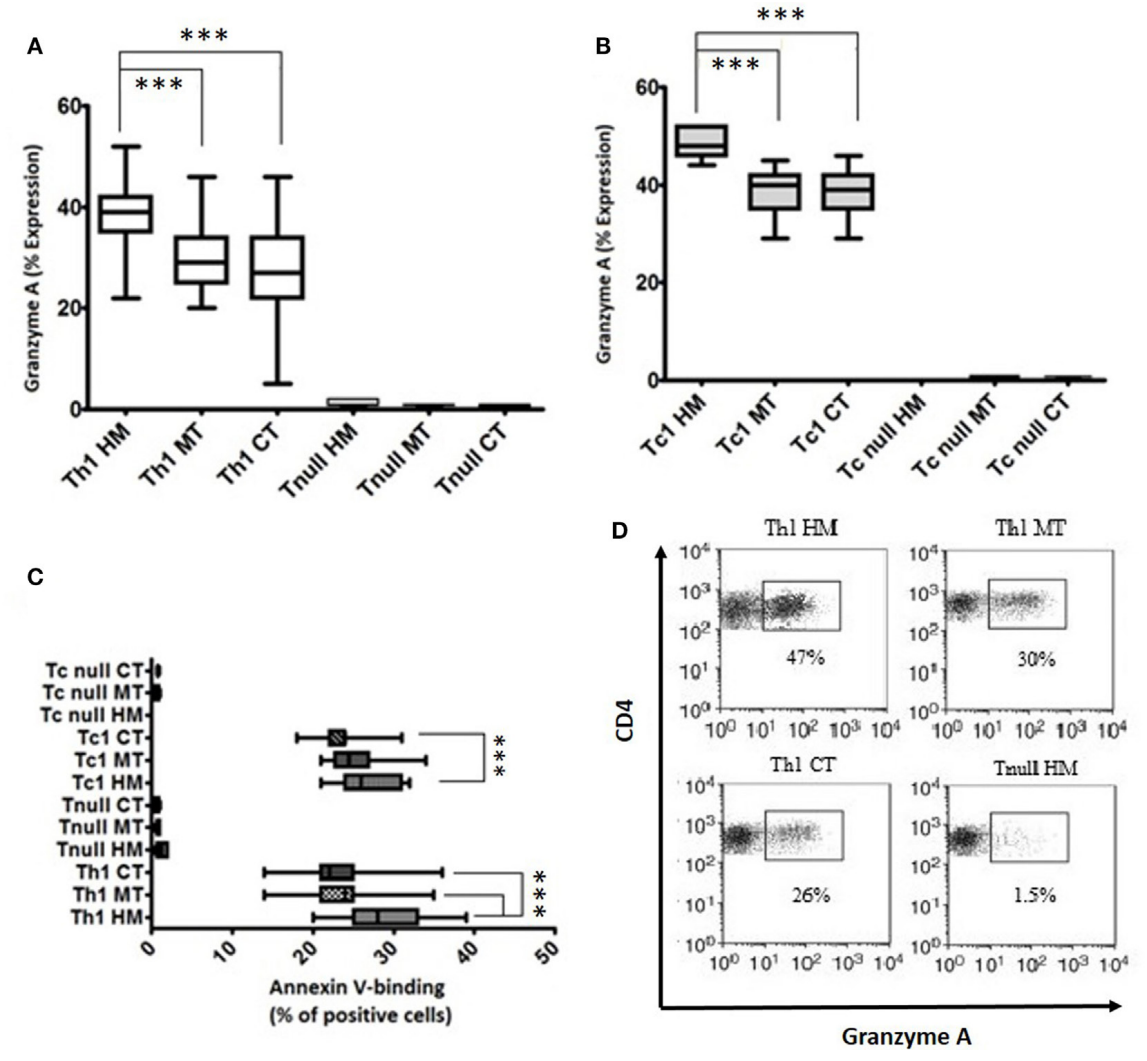
Cytotoxicity profile of T cell clones (Tcc) isolated from the different tumor sites. The granzyme A expression of CD4^+^ **(A)** and CD8^+^ **(B)** Tcc, and the percentage of annexin V-binding cells **(C)** were assessed according to the manufacturer’s instructions and analyzed on a FACSCanto cytofluorimeter (BD Biosciences) using FACSDiva software. The error bars represent the SD. Statistical analyses were calculated using one way ANOVA and Turkey’s *post hoc*. The asterisks (*) represent *p*-values, *p < 0.05, **p < 0.01, ***p < 0.001. **(D)** A representative dot plots indicating the granzyme A expression (percentage) of CD4 + Th1 and Tnull Tcc isolated from healthy mucosa (HM), marginal tumor (MT), and central tumor (CT).

Using the P815 cells as targets, we have assessed the cytolytic potential of Tcc (CD4^+^ and CD8^+^) with Th1 profile or Tnull; we have observed that all the Th1/Tc1Tcc showed an expression of granzyme A around 40% or slightly more, especially the CD8^+^ Tcc (Figures 5A,B). While, as expected, the Tcc with Tnull profile, as CD4^+^ (Figure 5A) as CD8^+^ (Figure 5B), are unable to kill the target cells.

Of note, the cytotoxic activity was different in the various Th1/Tc1 clones, isolated from the different tumor sites. As expected, the expression of granzyme A was significantly higher in Th1/Tc1Tcc isolated from HM compared to that released from the tumor tissue counterparts (Figures 5A,B,D).

In addition, we have observed that the intra-tumoral Tcc with Th17 profile were able to kill the target cells, only if they co-produced IFN-γ (data not shown).

Finally, we have assessed the ability of Tcc to induce apoptosis. All the Tnull Tcc displayed a very low percentage of AnnexinV-binding cells (<1%). Only the Tcc (as CD4^+^ as CD8^+^) with Th1 profile displayed a higher percentage. In addition, similar to what observed for the cytotoxicity, the Th1/Tc1 Tcc isolated from HM showed a major aptitude to kill the target inducing apoptosis (Figure 5C).

### Cytofluorimetric Assessment of Intra-Tumoral T Cells’ Distribution in CRC Patients

**Figure 6:**
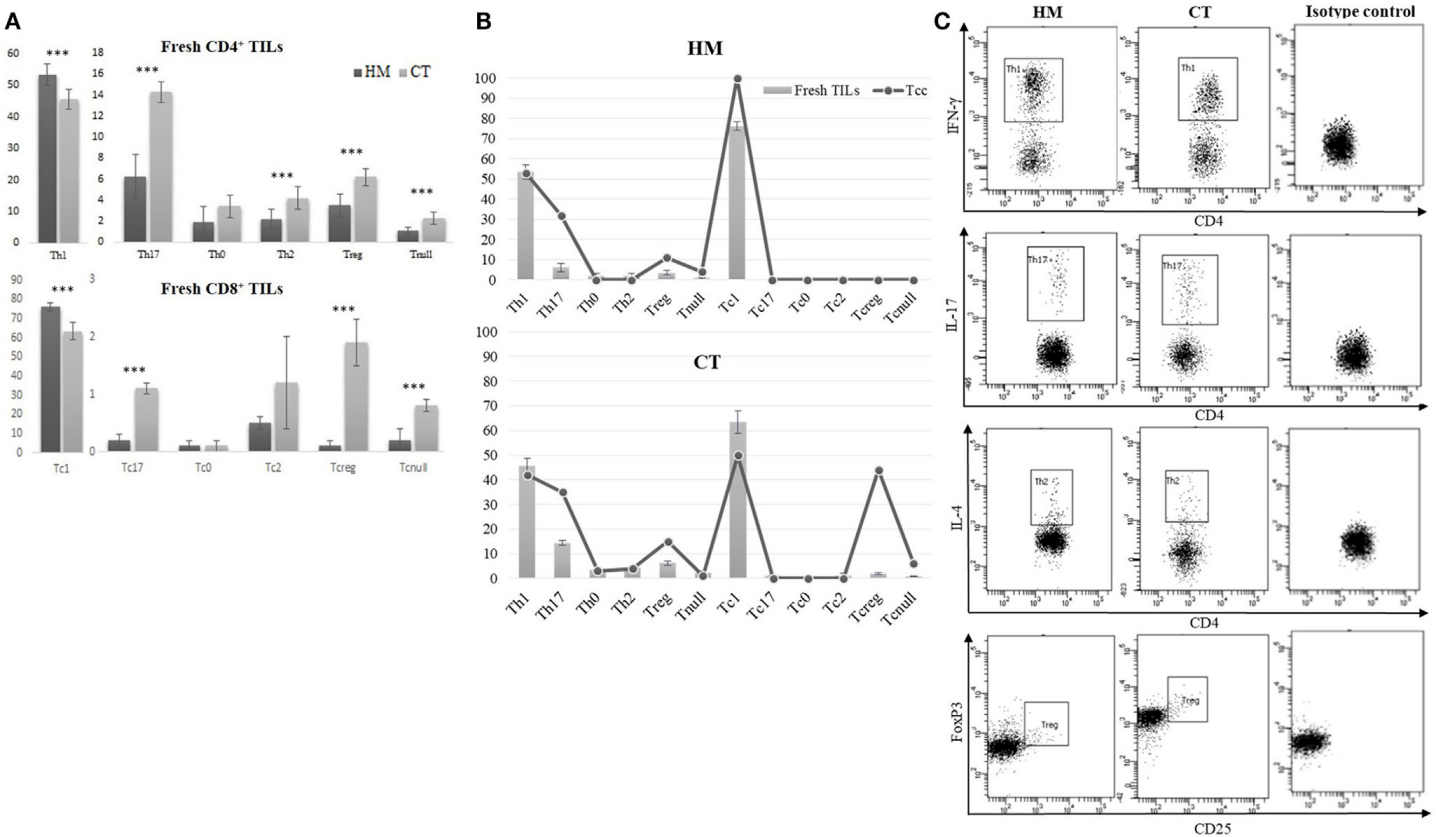
Cytofluorimetric analysis of fresh TILs isolated from tissue specimens. **(A)** Different distribution of fresh CD4^+^ and CD8^+^ TILs subsets, isolated from the healthy mucosa (HM) of 30 colorectal cancer (CRC) patients and the central tumor (CT) of 20 (out of 30). Results are reported as percentages (mean ± SD) and statistical analysis was performed using the Student’s *t*-test. The asterisks (***) represent a *p* < 0.001. **(B)** Comparison of CD4^+^ and CD8^+^ T cell subsets’ distribution, obtained by analysis of fresh TILs and Tcc obtained by cloning, in HM and CT of CRC patients. **(C)** A representative cytofluorimetric analysis of fresh T helper subsets (Th1, Th17, Th2, Treg) observed in HM and CT of the same CRC patients.

From the dissociated HM of all CRC patients and the CT tissue of 20 (out of 30), we performed a cytofluorimetric analysis of “fresh” infiltrating T cells (Figures 6A,C). As shown in Figure 6A, the CD4^+^ TILs isolated from CT contained a smaller percentage of Th1 (45.61 ± 3.11 vs 53.54 ± 3.53; *p* < 0.0001) and higher percentages of Th17 (14.30 ± 1.12 vs 6.21 ± 2.18; *p* < 0.0001), Treg (6.25 ± 0.81 vs 3.56 ± 1.12; *p* < 0.0001), Th2 (4.26 ± 1.12 vs 2.21 ± 0.90; *p* < 0.0001), and Tnull cells (2.31 ± 0.66 vs 1.12 ± 0.32; *p* < 0.0001) than TILs isolated from HM. In a similar way, CT contained a smaller percentage of Tc1 and a higher percentage of Tc17, Tcreg, and Tcnull than HM. Moreover, both CD4^+^ and CD8^+^ (Figure 6B) subsets of the TILs’ population mirror the distribution of Tcc obtained from the HM and CT.

### Evaluation of Circulating T Cell Subsets in CRC Patients

**Figure 7:**
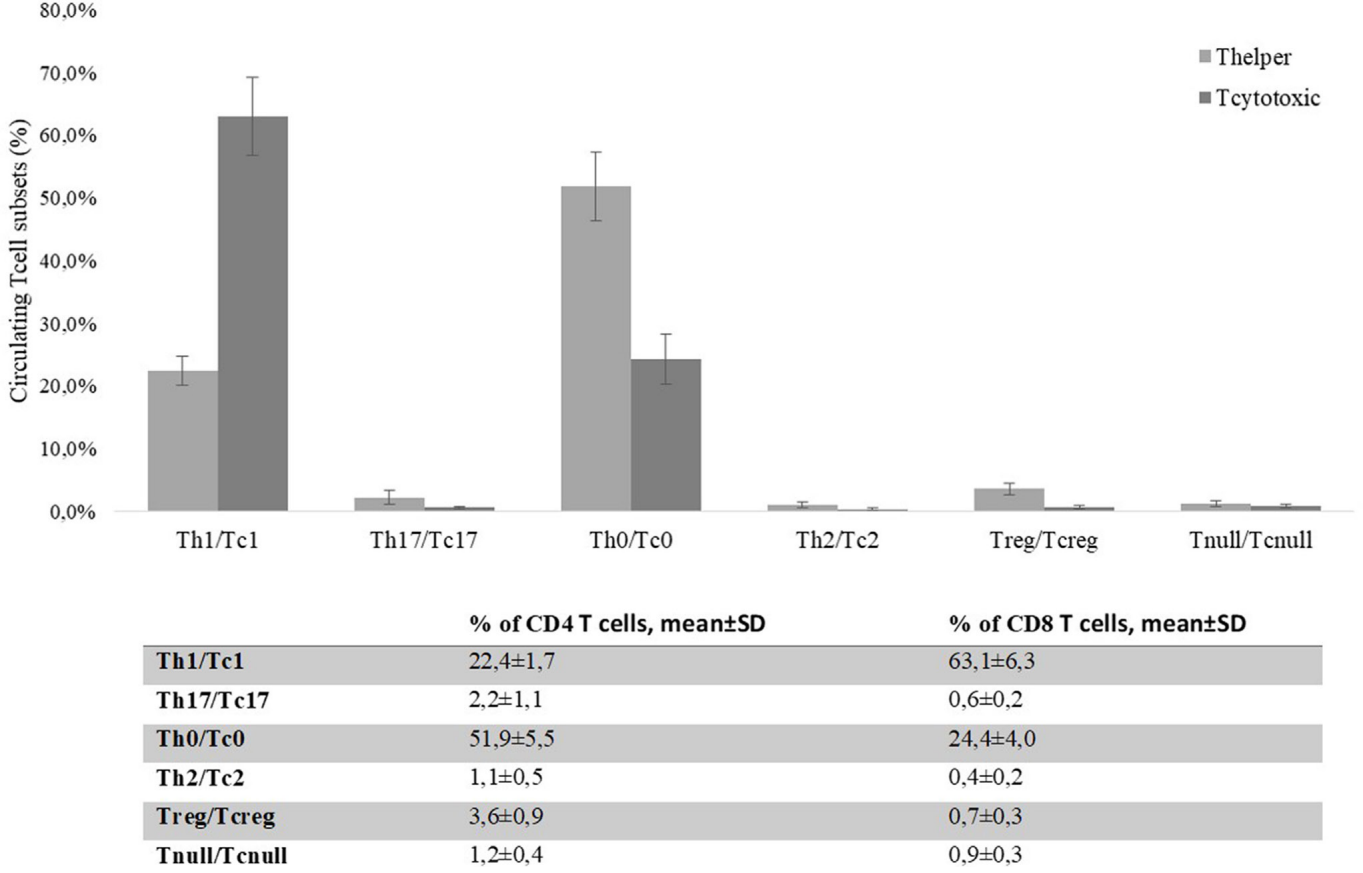
Percentages of CD4^+^ and CD8^+^ circulating T cells’ subsets present in colorectal cancer patients’ blood on the day of the surgery (*T* = 0). Evaluation of the T cell subsets was performed by flow cytometry basing on specific markers’ expression. T helper and T cytotoxic subsets are reported as percentages (mean ± SD) of total CD4^+^ or CD8^+^ T cells, respectively.

**Figure 8:**
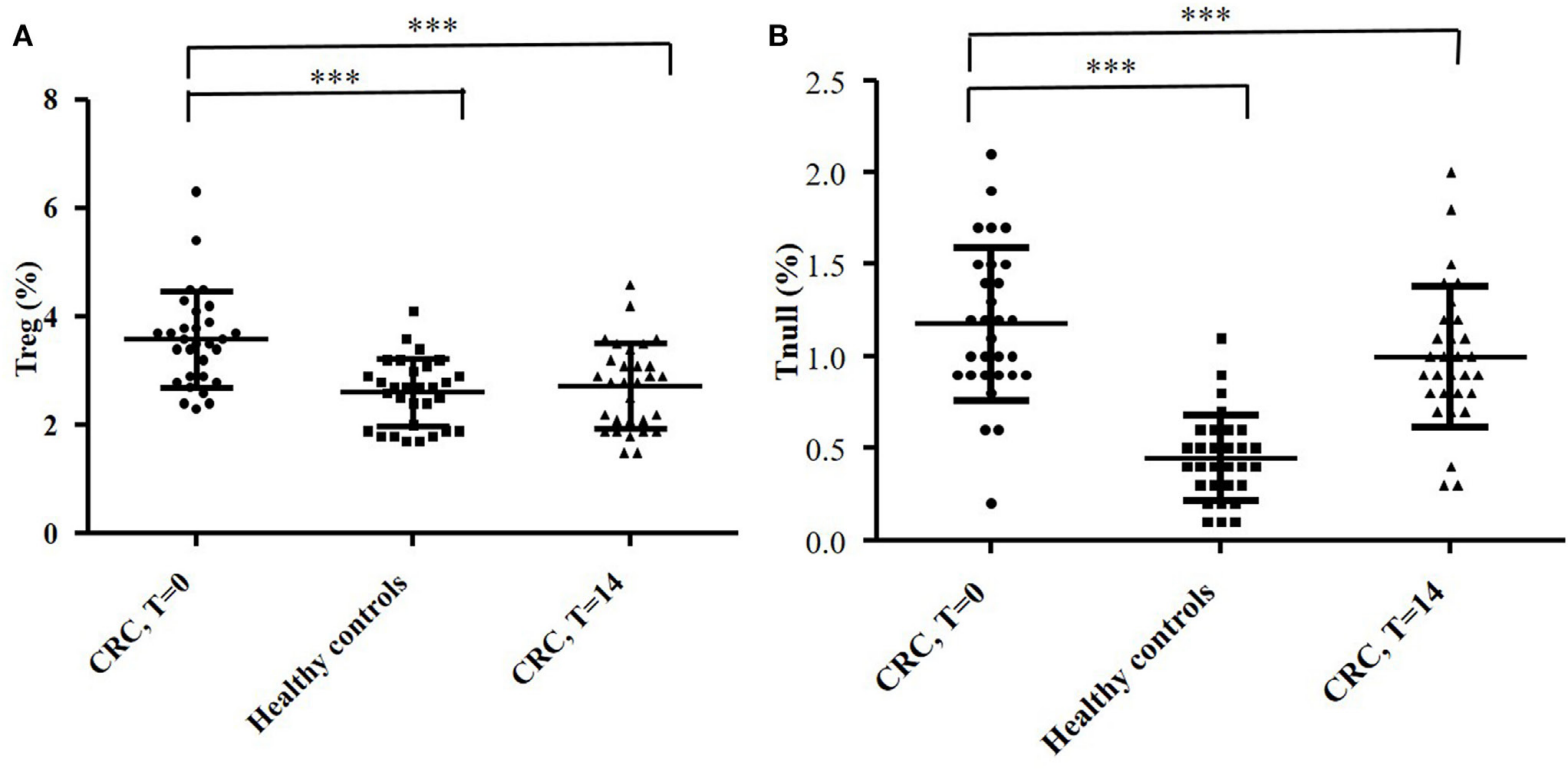
Evaluation of circulating CD4^+^ Treg **(A)** and Tnull **(B)** cells in blood samples of 30 colorectal cancer (CRC) patients and 30 healthy subjects. The evaluation of the T cells’ subsets was performed by flow cytometry (basing on the expression of specific markers) and at different time points: the day of the surgery (*T* = 0) and 2 weeks after (*T* = 14). The error bars represent the SD. Statistical analyses were calculated using one-way ANOVA and Turkey’s *post hoc*. The asterisks (*) represent *p*-values, *p < 0.05, **p < 0.01, ***p < 0.001.

By flow cytometry, we analyzed the distribution of T cell subsets in the peripheral blood of CRC patients. 61 ± 4.3% of peripheral T cells was positive for CD4^+^, while 15 ± 2.2% was CD8^+^. The mean percentages of CD4^+^ and CD8^+^ circulating T cell subsets obtained from the 30 CRC patients’ blood samples (collected at *T* = 0) are reported in Figure 7. The percentage of circulating Tregs registered in the CRC patients (3.58 ± 0.88) was significantly (*p* < 0.0001) higher compared to that of healthy controls (2.59 ± 0.62, Figure 8); anyway, multiple regression analysis did not show any correlation between preoperative circulating Tregs’ percentage values and age, sex, serum CEA level, and degree of histologic differentiation. The percentage of circulating CD4^+^ Tnull in CRC patients (1.17 ± 0.41) was significantly (*p* < 0.0001) higher than healthy controls (0.45 ± 0.23).

In addition, the percentage of circulating Tregs dropped significantly at *T* = 14 (2.72 ± 0.79) (*p* < 0.0001) and overlapped the values recorded in healthy controls (Figure 8A); conversely, the percentage of Tnull diminish, but not significantly, in the postoperative (0.99 ± 0.37) (Figure 8B). Of note, we have observed that the CRC patients with the highest percentage of circulating Tregs were those who exhibited a greater number of intra-tumoral Tregs, in particular patients 2, 4, 13, and 25.

### Assessment of Tregs Function

Finally, in a reduced number (*n* = 22) of patients, we obtained a suitable number of Tregs for the functional assays. First, by flow cytometry, we assessed the purity of the CD4^+^CD25^high^ T cell population, which was, in all cases, >90% (mean ± SD 93.6 ± 1.5; median 93.3; range 91.6–95.7). In a 1:1 ratio with Teff, Tregs were shown to inhibit growth of polyclonally stimulated Teff as demonstrated by tracking of VPD450 incorporation in lymphocytes by means of flow cytometry (data not shown). Therefore, the CD4^+^CD25^+^CD127^dim/−^ T phenotype was confirmed to belong to a T cell subset with immunosuppressive features.

## Discussion

In this study, we analyzed the peripheral and intra-tumoral T cell response in patients with CRC. To evaluate the intra-tumoral immune response, we isolated and characterized TILs from three different sites: the CT, the MT, and surrounding HM. In all three sites, the analysis of TIL clonal progeny has shown a large predominance of CD4^+^ T cells compared to the CD8^+^ T cell population. While the CD4^+^Tcc had a similar distribution in the three different sites, CD8^+^ Tcc were notably numerous on cancer tissue than HM. The TIL cytokine profile demonstrated that most CD4^+^ and CD8^+^ Tcc predominantly produced IFN-γ and a considerable fraction of CD4^+^ Tcc, but none of the CD8^+^ Tcc, also secreted IL-17 alone or in combination with IFN-γ. For their cytotoxic activity, Th1/Tc1 cells are critical in the antitumor response (11), while the role of IL-17 in tumorigenesis has not been clarified (37). Various studies reported the ability of Th17 to promote cancer growth; in particular, in CRC, Galon and collaborators found that a high infiltration of IL-17^+^ T cells was negatively correlated with patient’s prognosis (20), and Sharp et al. demonstrated that advanced stage colon cancer was associated with elevated Th17-associated cytokines (14). Anyway, endogenous IL-17 or/and Th17 cells may play a protective role in tumor immunity (13, 38, 39); for example, levels of tumor-infiltrating Th17 cells were reduced in advanced ovarian cancer patients, which appeared to correlate with a positive outcome (39). In pancreatic cancer, we have demonstrated the effectors functions of antigen specific Th1/Th17 Tcc (40) supporting the theory that, when Th17 turn into IFN-γ-expressing Th17 (Th17/Th1 cells), they can contribute to protective antitumor immunity, eradicating tumor cells through production of IFN-γ, or inducing Th1-type chemokines and stimulating CXCL9 and CXCL10 production to recruit effector cells to the tumor microenvironment (39, 41, 42). Regarding CRC, Amicarella and collaborators revealed a positive contribution of Th17 cells to beneficial antitumor immune responses in CRC and underline their pleiotropic function resulting from the production of a broad spectrum of cytokines and chemokines beyond IL-17 (13). Therefore, to confirm their effector functions, we tested the cytotoxic properties of the IFN-γ producing Tcc, both Th1/Tc1 and Th1/Th17, isolated from CRC patients. We found that they were all able to kill cancer cells, but the percentage of effectors T cells decreased in cancer tissue than in HM and, interestingly, the amount of IFN-γ produced by CD4^+^ Tcc isolated from HM was significantly higher compared to that produced by Th1 Tcc generated from both MT and CT. These data highlight an impairment of the antitumor immune response in the tumor sites. Moreover, the cytotoxic activity was different in the various Th1/Tc1 clones, isolated from the different tumor sites: in fact, the expression of granzyme A was significantly higher in Th1/Tc1 Tcc isolated from HM compared to that released from the tumor tissue counterparts, suggesting that the tumor microenvironment negatively influences the cytolytic activity of T lymphocytes reactive to tumor cells.

Another interesting data are the presence in the cancer tissues, but not in HM, of CD4^+^ Tcc able to secrete IL-4 alone or in combination with IFN-γ, showing a Th2/Th0 profile; notoriously, these cells can favor cancer progression and particularly in CRC, it is reported that Th1 cell predominance is correlated with a good prognosis, whereas a high proportion of Th2 cells is correlated with a worse prognosis (43, 44). Anyway, the most striking data shown by our analysis are that the tumor is infiltrated by a large amount of Tcc with regulatory or “non-functional” profile. In fact, the 13% of CD4^+^ Tcc and the 49% of CD8^+^ Tcc did not display effector properties.

We have isolated CD4^+^ neT Tcc from all the three sites, and the 11% of them have proved to be Tregs, while the 2% were Tnull. The Tregs’ percentage was higher in the CT than in the surrounding HM, and the percentage of Tnull was slightly higher in HM than in CT and MT. About the CD8^+^ neT Tcc, the majority showed a Tcreg profile (43%) while the 6% were Tcnull, with a similar distribution in MT and CT; none CD8^+^ neT Tcc was isolated from the HM. So, the CRC tissue is massively infiltrated by CD8^+^ T cells, but many of them (about half of the isolated Tcc) have a regulatory profile or are anergic, i.e., not able to produce cytokines with anticancer role [primarily, IFN-γ (45)].

We demonstrated the functional anergy of CD4^+^ and CD8^+^ Tnull clones by their inability to lyse target cells. Dysfunctional CD4^+^ and CD8^+^ T cells have been defined in both cancer patients and experimental models (16, 17, 46). These cells include exhausted, anergic, and regulatory/senescent T cells. Exhausted T cells are a persistent T cell population characterized by the low cytokine production, reduced cytotoxic activity, and reduced proliferative potential (47, 48). Anergy refers to a hyporesponsive state of impaired IL-2 production and proliferation, resulting from inefficient costimulation and/or high co-inhibitory signaling or from partial or chronic TCR stimulation (17). T cell exhaustion represents a distinct but reversible T cell fate in the context of antitumor immune responses and moreover, many studies suggest that immunosuppressive mechanisms in the tumor microenvironment are capable to promote an anergic phenotype (49). In CRC patients, a recent study demonstrated the functional impairments in the cytokine production and proliferation of T cells infiltrating tumor, probably related to T cell exhaustion (15). Similarly, populations of Tregs have been largely identified in cancer, and are usually considered able to protect cancer cells from antitumor immunity. In fact, they are capable to block the T-cell-mediated immune response against human tumors (50), including CRC (51), and the presence of Tregs is correlated with a poor prognosis in various cancers (27, 52, 53). However, recent conflicting reports show that high expression of Tregs is correlated with a good prognosis, especially in CRC (10).

The Tregs’ protective role may depend on their control of inflammation associated with neoplastic transformation and cancer progression (54–57). We, in agreement and supported by other study, believe that Tregs can play protective roles prior to cancer initiation in “inflammation-prone” cancers but, after the tumor establishment, the Tregs can be co-opted by tumors assuming a pro-tumorigenic role (58). In fact, an accumulation of highly suppressive and activated FoxP3^+^ Tregs in the tumor tissue of CRC patients has also been reported to correlate with tumor progression (59–61). Moreover, today, it is well known that various Tregs subsets exist, which differ in their suppressive ability, i.e., “effectors Tregs” or “non-Tregs” (33). About them, a recent study showed that CRC patients can be separated into two groups: (i) with tumors infiltrated predominantly by suppression-competent effectors Tregs and (ii) with tumors infiltrated with a sizable fraction of Foxp3^+^ non-Tregs in addition to effectors Tregs (62). In the latter group, Foxp3^+^ non-Tregs secrete inflammatory cytokines and the cytokine production is correlated with expression of TGF-β and IL-12 genes in the cancer cells. In this group, high Foxp3 gene expression shows significantly better prognosis than low Foxp3 gene expression. By contrast, in the group with tumors infiltrated predominantly by effectors Tregs, high Foxp3 gene transcription indicates poor prognosis compared with low Foxp3 transcription. Hence, the Tregs’ subsets can play a contrasting role in tumor immunity and to identify suppressive Tregs it is critical to test their immunosuppressive properties through functional assays. As reported above, in our study, we have observed a considerable number of intra-tumoral Tregs in CRC patients, and interestingly their prevalence was higher in cancer tissue than in surrounding HM, especially regarding the CD8^+^ Tcc, suggesting a Tregs’ induction and accumulation within the tumor microenvironment. Notably, as further confirmation of these *ex vivo* data, we also documented a similar trend *in vivo*. In fact, the cytofluorimetric analysis of fresh TILs subsets’ distribution mirrors the Tcc subpopulations, obtained by the cloning approach. Also, we have evaluated the presence of Tregs in the peripheral blood of CRC patients and we have observed a higher percentage of preoperative circulating Tregs (Table 1) in patients that in healthy volunteers, even if the circulating Tregs percentage values did not correlate with patients’ clinical parameters. Interestingly, we have observed that the CRC patients with the highest percentage of circulating Tregs were those who exhibited a greater number of intra-tumoral Tregs showing that the peripheral immune response seems to mirror them of intra-tumoral site.

In more than 70% of the CRC patients, we demonstrated the immunosuppressive properties of the circulating Tregs, confirming their ability to impair the antitumor immunity by reduction of effectors T cells proliferation. Finally, evaluating the percentage of circulating Tregs 1 week after the surgical removal of the tumor mass, we observed, in line with previous data (36) a significant reduction of the circulating Tregs’ values that goes down to the values recorded in healthy controls, confirming the cancer role in the modulation of the immune system, especially supporting a Tregs-mediated immunosuppression. As additional confirmation of the immune modulating role of cancer cells, in the peripheral blood of CRC patients, we found the presence of CD4^+^ Tnull cells, whose percentage was significantly higher than that in healthy controls.

In summary, our data show the impairment of the antitumor immunity in the context of the CRC microenvironment, documented by the weakening of the effector functions of Tcc from HM to colon cancer tissues, and the increase of T lymphocytes’ subsets (Th2/Th0/Tregs/Tnull) that can promote tumor progression. In particular, we suppose that the ratio Teff/Tnet (Treg + Tnull) can play a crucial role in the impairment of antitumor immunity, and further studies could be planned to evaluate this hypothesis. Finally, therapies aimed to favor antitumor immunity must be designed to deplete the “effectors” Tregs as well as enhancing effectors functions of Tcc with anticancer properties.

## Ethics Statement

The study was reviewed and approved by AOUC Careggi Institutional Review Board (Prot. 2010/0012462). The name of the local ethical committee is “Comitato Etico Area Vasta Centro.” All study participants, or their legal guardian, provided informed written consent prior to study enrollment in compliance with national legislation and the Code of Ethical Principles for Medical Research Involving Human Subjects of the World Medical Association (Declaration of Helsinki).

## Author Contributions

EN, FR, and AA conceived and designed the study, and drafted the paper. EN, FR, ER, GN, and GE acquired experimental data. AT, MR, FM, MM, PB, and FC were involved in enrollment and obtaining clinical data of patients. EN, DP, FR, and AA analyzed and interpreted data. AA, GN, and DP critically revised the paper.

## Conflict of Interest Statement

The authors declare that the research has been conducted in the absence of any commercial or financial relationships that could be construed as a potential conflict of interest.

## Abbreviations

CRC, colorectal cancer; TILs, tumor-infiltrating lymphocytes; Tcc, T cell clones; Tregs, T regulatory cells; neT Tcc, non-effectors T cell clones; Teff, T effectors cells.
